# Chemical Composition, Antibacterial and Antioxidant Activities of Essential Oil from *Centipeda minima*

**DOI:** 10.3390/molecules28020824

**Published:** 2023-01-13

**Authors:** Fan Su, Gan Yang, Datong Hu, Chen Ruan, Jing Wang, Yingying Zhang, Qingjun Zhu

**Affiliations:** 1 School of Pharmacy, Shandong University of Traditional Chinese Medicine, Jinan 250355, China; 2 Shandong Academy of Pharmaceutical Sciences, Jinan 250098, China; 3 School of Traditional Chinese Medicine, Shandong University of Traditional Chinese Medicine, Jinan 250355, China; 4 Innovation Research Institute of Traditional Chinese Medicine, Shandong University of Traditional Chinese Medicine, Jinan 250355, China

**Keywords:** essential oil, *Centipeda minima*, chemical composition, antibacterial, antioxidant, GC-MS

## Abstract

This study elucidated the chemical composition of essential oil from *Centipeda minima* (EOCM) and its antibacterial and antioxidant activities with two chemical monomers thymol and carvacrol. The main chemical composition of EOCM, analyzed by GC-MS, were trans-chrysanthenyl acetate, thymol, aromadendrene and β-caryophyllene. In the screening of antibacterial activity against *S. aureus*, two monomers with antibacterial activity were obtained: thymol and carvacrol. The MIC of EOCM, thymol and carvacrol were 0.625 mg/mL, 0.156 mg/mL and 0.156 mg/mL, respectively. The experimental results were shown that three drugs could inhibit the growth of *S. aureus* and inhibit the formation of biofilm by changing the permeability of cell membrane and interfering with the metabolic activities in bacteria. The scavenging effects of the three drugs on DPPH radical and hydroxyl radical showed that the antioxidant effect of the three drugs was EOCM > carvacrol > thymol.

## 1. Introduction

Natural drugs have been used for centuries; plant-derived natural drugs especially have many beneficial effects, including antibacterial, antioxidant, anti-inflammatory, anti-tumor, anti-virus and other effects. Natural drugs have a wide range of sources and are relatively safe. Different biological activities can often be obtained through different treatment methods, and the effect is greatly enhanced.

Essential oil is a general term for a group of volatile oil-like components with aromatic odors, which can be distilled with water vapor and are not miscible with water [1]. Essential oil is a mixture with complex components. Most of the essential oils have an aromatic odor, which contain terpenoids in addition to aliphatic compounds, aromatic compounds and oxidation derivatives. In recent years, the research on natural drugs has gradually increased. Essential oil widely exists in plants, has a wide range of sources and plays significant anti-bacterial and anti-viral roles [2,3,4,5].

The *Centipeda minima*, whole herb of the Compositae plant *Centipeda minima* (L.) A. Braun et Aschers, is mainly produced in Jiangsu, Zhejiang, Guizhou, Guangdong, Guangxi, Fujian, Anhui and other places [6]. It has the functions of dispersing cold, relieving the nose, relieving cough, detoxification, etc. [7] It is mainly used to treat cold headaches, stuffy nose, coughs, runny noses and other symptoms. The research shows that *Centipeda minima* has anti-bacterial, anti-inflammatory, anti-allergic and other pharmacological effects [8,9,10]. The main chemical components are essential oils, flavonoids, terpenoids and steroids [11].

*Staphylococcus aureus* (*S. aureus*) is a common food=borne pathogen, which can produce enterotoxin under appropriate conditions and cause food poisoning [12]. The proportion of food poisoning incidents caused by *S. aureus* in food-borne microbial food poisoning incidents reached a quarter of the total, becoming the third largest microbial pathogen after *Salmonella* and *Vibrio Parahaemolyticus*. Bacterial infection seriously endangers public health, and most bacteria will produce biofilm. Bacterial biofilm is a kind of membrane sample formed by a large number of bacteria gathered by bacteria adhering to each other and secreting a large number of macromolecular substances. Compared with planktonic bacteria, the drug resistance of mature biofilm is significantly increased, which is not conducive to the treatment of bacterial infection [13]. Antibiotic treatment is the most conventional method for bacterial infection at present, but bacteria that have been treated with antibiotics easily develop drug resistance, which reduces the efficacy and cannot produce the desired effect [14]. Therefore, to find more safe and effective drugs is the direction of existing research.

In this study, the chemical components of EOCM were analyzed, and some of these chemical components were screened for antibacterial activity. Two kinds of monomers with antibacterial activity were obtained, and their antibacterial mechanisms were explored. The antioxidation of EOCM and two monomers were also determined.

## 2. Results

### 2.1. Chemical Component Analysis of EOCM

The chemical composition of the essential oil was analyzed by GC-MS, and the results are shown in Table 1. Sixty compounds were obtained by searching the database, accounting for 88.15% of the total essential oil. The main components with high content are trans-chrysanthenyl acetate (20.23%), aromadendrene (11.47%), thymol (5.69%) and β-caryophyllene (5.26%). The main compounds of EOCM are terpenoids and esters.

**Figure 1:**
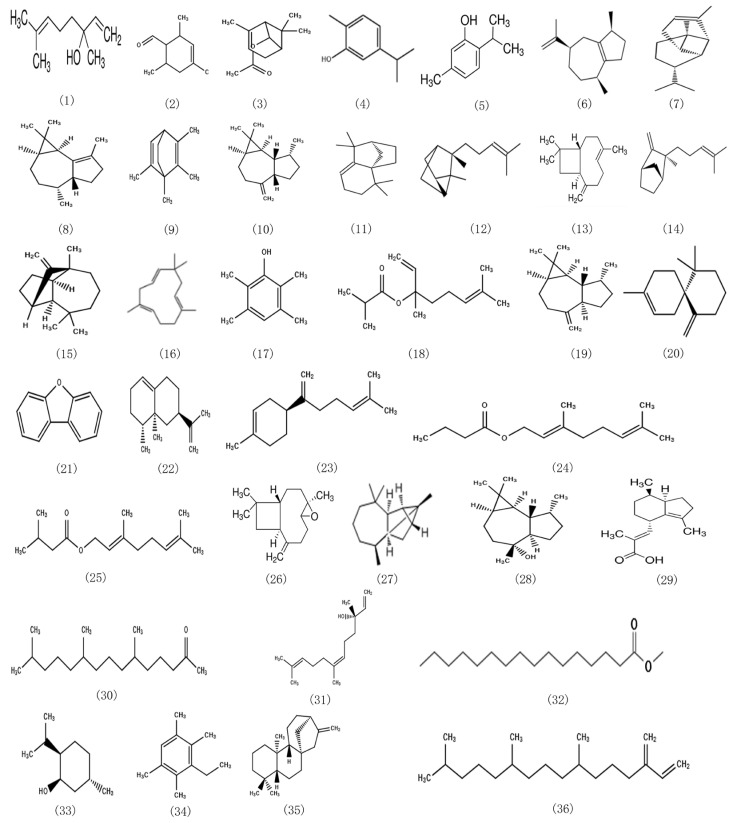
Structural formula of chemical components with content greater than 0.5% in EOCM.

The essential oil used for this analysis is obtained by steam distillation. In combination with the compound information obtained in Table 1 and the database retrieval results, the structural formula of the compound monomer with a relative content of more than 0.5% is summarized in Figure 1.

### 2.2. Determination of Antibacterial Activity

#### 2.2.1. MIC and MBC of EOCM to *S. aureus*

As shown in the Table 2, the MIC experiment shows that the EOCM has significant antibacterial activity at a concentration of 0.625 mg/mL, and two essential oil monomers, thymol and carvacrol, with significant antibacterial activity are obtained. Through determination, both monomers show strong antibacterial activity at a concentration of 0.156 mg/mL. The results showed that the antibacterial activity of thymol and carvacrol was stronger than that of EOCM. The reason may be that the content of monomer in essential oil was lower than that of monomer, and the antibacterial activity of EOCM was reduced.

#### 2.2.2. Growth Curve

**Figure 2:**
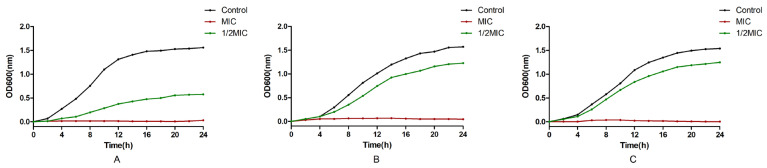
Effect of EOCM (**A**), thymol (**B**) and carvacrol (**C**) on the growth of *S. aureus*. Error bars represent the standard deviations (SD) of the means from three biological replicates (*n* = 3) in an individual experiment.

The effect of EOCM, thymol and carvacrol on the growth of *S. aureus* is shown in Figure 2. After the treatment of *S. aureus* with three drugs, the growth of bacteria was affected. After 4 h of drug treatment, compared with the control group and the 1/2 MIC drug group, the growth of bacteria in the MIC treatment group began to be inhibited, which was significantly different from the control group.

#### 2.2.3. Effect on the Permeability of Bacterial Cell Membrane

**Figure 3:**
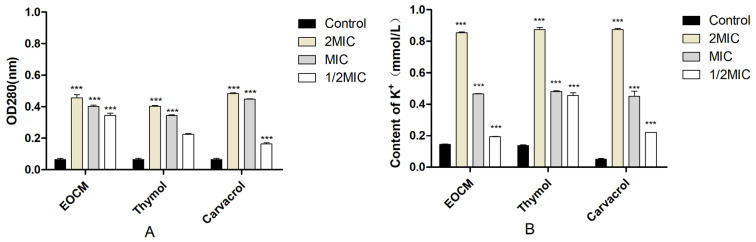
(**A**) is the release of nucleic acids from *S. aureus* treatment with EOCM, thymol and carvacrol. (**B**) is the content of K^+^ from *S. aureus* treatment with EOCM, thymol and carvacrol. Error bars represent the standard deviations (SD) of the means from three biological replicates (*n* = 3) in an individual experiment. ***, results were significantly different from those of control by a *t* test (*p* < 0.001).

As shown in Figure 3A, the absorbance of the three drug groups at 260 nm increased with the increase of concentration after 1 h of incubate with the drug, which was significantly different from the control group (*p* < 0.05). The results in Figure 3B show that the extracellular K^+^ concentration of the three experimental groups after incubate with the drug is significantly higher than that of the control group (*p* < 0.05). Combined with nucleic acid substances such as extracellular DNA and K^+^ content, the phenomenon of the increase after drug treatment can explain that EOCM, thymol and carvacrol can change the permeability of the *S. aureus* cell membrane, leading to the outflow of intracellular substances, thus inhibiting the growth and reproduction of bacteria.

#### 2.2.4. Determination of Alkaline Phosphatase (AKP) Activity

**Figure 4:**
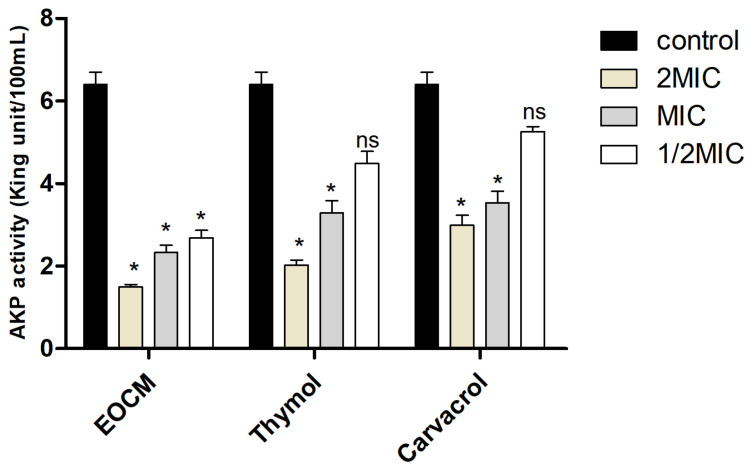
AKP activity from *S. aureus* treatment with EOCM, thymol and carvacrol. Error bars represent the standard deviations (SD) of the means from three biological replicates (*n* = 3) in an individual experiment. *, results were significantly different from those of control by a *t* test (*p* < 0.05). ns, results were no significantly different from those of control by a *t* test (*p* > 0.05).

AKP is a kind of phosphate hydrolase which exists in a large number of microorganisms and plays an irreplaceable role in the phosphate metabolism. AKP exists between the cell wall and cell membrane. When the integrity of the cell wall or the cell membrane is damaged, AKP will leak into the incubate medium, reducing the AKP activity in bacteria. In this experiment, the effect of EOCM, thymol and carvacrol on AKP activity was determined to reflect the damage to the cell wall of *S. aureus*. As shown in Figure 4, after 2 h of drug treatment, when the drug concentration was 2MIC and MIC, the AKP activity of the three experimental groups was significantly lower than that of the control group (*p* < 0.05), EOCM has the most significant effect. The results showed that EOCM, thymol and carvacrol could damage the cell wall of *S. aureus* to some extent.

#### 2.2.5. Determination of Total ATPase Activity in and out of the Bacteria

**Figure 5:**
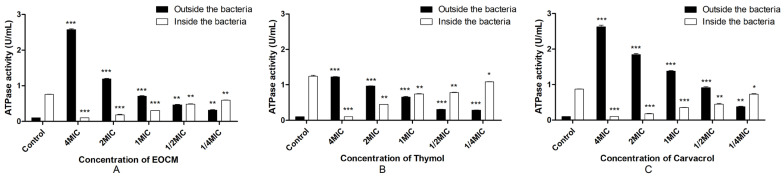
ATPase activity from *S. aureus* treatment with EOCM (**A**), thymol (**B**) and carvacrol (**C**). Error bars represent the standard deviations (SD) of the means from three biological replicates (*n* = 3) in an individual experiment. ***, results were significantly different from those of control by a *t* test (*p* < 0.001). **, results were significantly different from those of control by a *t* test (*p* < 0.01). *, results were significantly different from those of control by a *t* test (*p* < 0.05).

As shown in Figure 5, the results showed that compared with the total ATPase content in and out of the bacteria without drug treatment, the ATPase activity inside the bacteria is significantly reduced (*p* < 0.05) after treatment with EOCM, thymol and carvacrol, and the ATPase activity outside the bacteria is significantly increased (*p* < 0.05). Combination with the change of cell membrane permeability and the decrease of ATPase activity inside *S. aureus*, EOCM, thymol and carvacrol can affect the ATPase activity of *S. aureus* and inhibit the metabolic activity of *S. aureus*, thus inhibiting the growth of *S. aureus*.

### 2.3. Determination of Anti-Biofilm Activity

**Figure 6:**
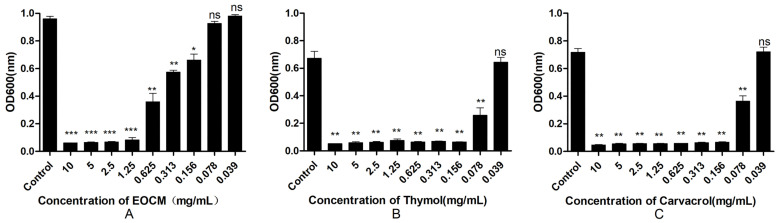
Effect of EOCM (**A**), thymol (**B**) and carvacrol (**C**) on the growth of biofilm. Error bars represent the standard deviations (SD) of the means from three biological replicates (*n* = 3) in an individual experiment. ***, results were significantly different from those of control by a *t* test (*p* < 0.001). **, results were significantly different from those of control by a *t* test (*p* < 0.01). *, results were significantly different from those of control by a *t* test (*p* < 0.05). ns, results were no significantly different from those of control by a *t* test (*p* > 0.05).

As shown in Figure 6A, EOCM has a significant anti biofilm effect on *S. aureus* at a concentration of 0.156 mg/mL (*p* < 0.05), and the effect increases with the increase of concentration, and the effect intensity reaches equilibrium at 1.25 mg/mL. As shown in Figure 6B, thymol has a significant anti biofilm effect on *S. aureus* at the concentration of 0.078 mg/mL (*p* < 0.05), and the effect increases with the increase of concentration, and the effect intensity reaches equilibrium at 0.156 mg/mL. As shown in Figure 6C, carvacrol has a significant anti biofilm effect on *S. aureus* at the concentration of 0.078 mg/mL (*p* < 0.05), and the effect increases with the increase of concentration, and the effect intensity reaches equilibrium at 0.156 mg/mL.

### 2.4. Scanning Electron Microscope (SEM) Analysis

**Figure 7:**
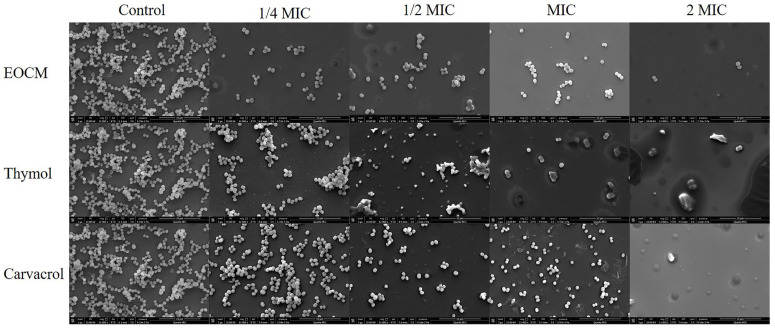
Effect of EOCM, thymol and carvacrol the growth of *S. aureus* biofilm and bacterial morphology (SEM, ×12,000).

As shown in Figure 7, after *S. aureus* was incubated with EOCM, thymol and carvacrol of different concentrations for 24 h, the aggregation and adhesion between bacteria decreased significantly. With the increase of drug concentration, bacteria began to disperse, the number of bacteria adhering to the glass gradually decreased, the morphology of bacteria began to change, and the phenomenon of cracking occurred. When the concentration reached 2 MIC, the glass plate can hardly see the complete bacterial structure. The reason may be that the bacteria cannot secrete adhesive substances after death, and most of the dead bacteria are washed away.

### 2.5. Confocal Laser Scanning Microscope (CLSM) Analysis

**Figure 8:**
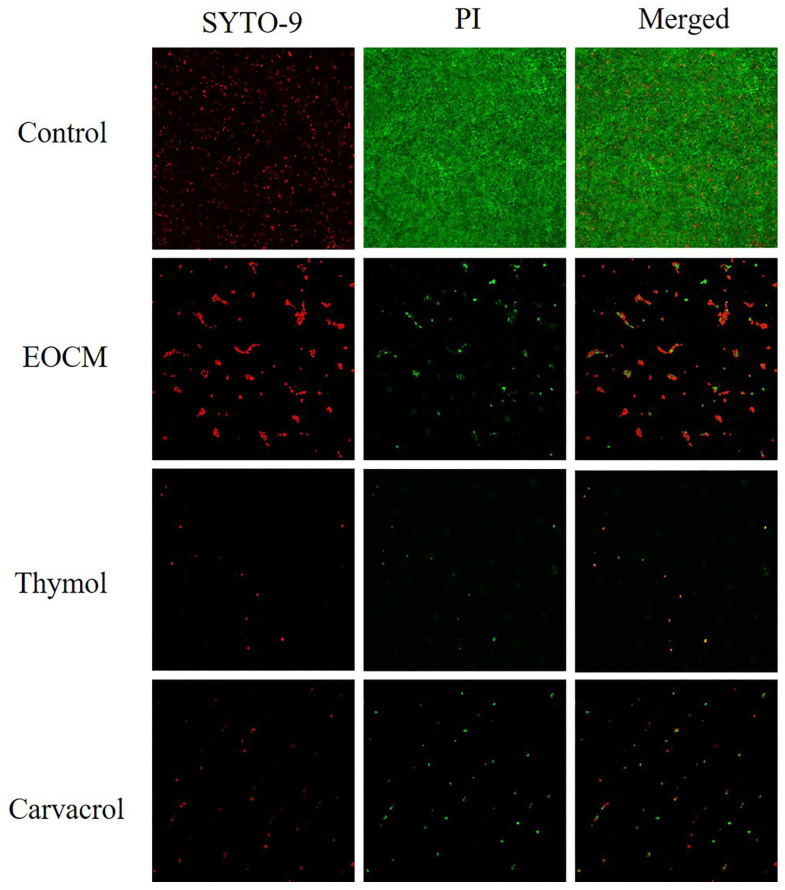
Effect of EOCM, thymol and carvacrol the growth of *S. aureus* biofilm under CLSM.

Fluorescent dyes used in the experiment include SYTO-9 and propidium iodide (PI). SYTO-9 is a green fluorescent nucleic acid dye that can penetrate the cell membrane, and can be used for RNA and DNA staining of living and dead eukaryotic cells, as well as Gram-positive and Gram-negative bacteria. PI is a nuclear staining dye that can release red fluorescence after binding with DNA double strands. PI could not penetrate the intact cell membrane, but could penetrate the damaged cell membrane of late apoptotic cells and dead cells, and make the nucleus red. As shown in Figure 8, after 24 h of drug incubate, compared with the control group, the number of bacterial biofilm and bacteria adhering to the surface of glass sheet in the three experimental groups decreased significantly, and the proportion of damaged bacteria and dead bacteria increased.

### 2.6. SDS-PAGE Electrophoresis of the Bacterial Proteins

**Figure 9:**
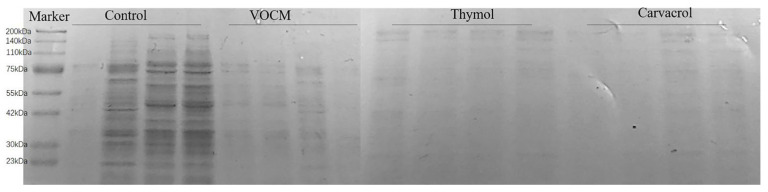
Effect of EOCM, thymol and carvacrol on bacterial protein synthesis. From left to right, they were divided into four groups: control, EOCM, thymol and carvacrol. Each group of protein bands from left to right were incubated with drugs for 3 h, 6 h, 9 h and 12 h, respectively.

Protein is the material basis of all life, which can maintain the normal metabolism and transportation of various substances in the body, and the enzymes that play a catalytic role in various biochemical reactions in cells and organisms are mainly proteins, so the level of protein expression can reflect the life state of cells. As shown in Figure 9, the *S. aureus* protein bands in the control group were basically stable and slowly deepened. After drug treatment, the protein bands of the three drug groups became more transparent, and the protein content was lower than that of the control group in the same period. It can be seen that EOCM, thymol and carvacrol can affect the protein synthesis of bacteria.

### 2.7. Antioxidant Activity

**Figure 10:**
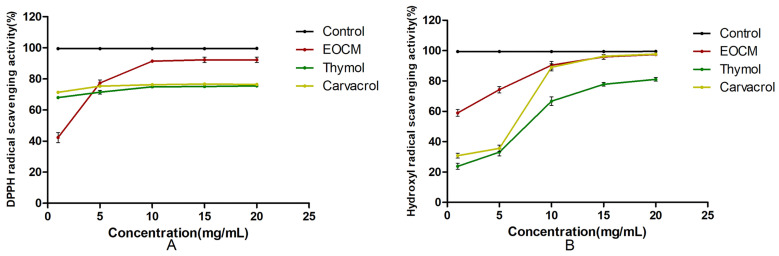
(**A**) is DPPH radical scavenging activity of EOCM, thymol and carvacrol. (**B**) is hydroxyl radical scavenging activity of EOCM, thymol and carvacrol. Error bars represent the standard deviations (SD) of the means from three biological replicates (*n* = 3) in an individual experiment.

As shown in Figure 10 and Table 3, within the concentration range of experimental samples, the scavenging rates of EOCM, thymol and carvacrol on DPPH radical and hydroxyl radical all enhance with the increase of drug concentration, indicating that the radical scavenging activity of the three drugs is dose dependent. The antioxidant activity of the three drugs was lower than that of V_C_, and EOCM had the strongest antioxidant activity.

## 3. Materials and Methods

### 3.1. Materials and Reagents

Fresh *Centipeda minima* plants were collected from Yantai, Guangxi Province, China. PBS were obtained from Servicebio (Wuhan, China). thymol and carvacrol were obtained from Macklin (Shanghai, China). AKP, K^+^ and ATPase kits were obtained from Nanjing Jiancheng Bioengineering Institute (Nanjing, China). Other chemical reagents were of analytical grade and purchased from Sinopharm Chemical Reagent Co., Ltd. (Shanghai, China). *S. aureus* was provided by Microbiology Department, Shandong University of Traditional Chinese Medicine (Jinan, China).

### 3.2. Extraction of Essential Oil

We took 500 g of dried *Centipeda minima* powder and placed it in a 10 L round-bottomed flask, added 5000 mL of distilled water to soak it for 2 h, heated it to keep it slightly boiling, and distilled for 6 h, collected the oil in the upper layer of the extractor, centrifuged the upper layer of the oil layer, added anhydrous sodium sulfate to dehydrate it, and then weighed it to obtain the essential oil from *Centipeda minima* (EOCM) [15].

### 3.3. Chemical Composition Analysis of EOCM

GC-MS analysis of EOCM was performed using GCMS-QP 2010 (Shimadzu, Japan) with a DB-5MS capillary column (30 m × 0.25 mm × 0.25 µm). The injection volume was 1 µL with a 10: 1 split ratio. Helium (99.999% purity) was used as the carrier gas at a flow rate of 1 mL/min. The temperature of the injector and MS transfer line were 230 °C and 250 °C, respectively. The initial temperature was kept at 50 °C, held for 2 min, and then it was gradually raised to 120 °C at 5 °C/min, and held for 2 min. Finally, it was raised to 250 °C at 10 °C/min and kept for 2 min. The temperature of the ion source was 200 °C. The mass-selective detector was operated in electron impact ionization (EI) mode with a mass scan range from *m*/*z* 45–500 at 70 eV.

The mass spectrometry data were initially identified using computer-automated comparison of the standard mass spectrometry database NIST. The chemical composition was manually analyzed based on the mass spectrometry information in conjunction with the relevant literature, and further confirmed by comparison with the literature values recorded in the NIST web database.

### 3.4. Determination of Antibacterial Activity

#### 3.4.1. Bacterial Liquid Incubate

The frozen *S. aureus* was inoculated in the MH solid medium for resuscitation, the *S. aureus* was picked up and inoculated in the MH liquid medium, incubated at 37 °C for 24 h, and the concentration of the bacterial solution was adjusted to 10^7^–10^8^ CFU/mL with the sterile MH liquid medium before the experiment.

#### 3.4.2. The MIC and MBC of EOCM to *S. aureus*

The minimal inhibitory concentration (MIC) of EOCM and its monomers was determined by the micro broth dilution method and screened the antibacterial activity of monomers [16]. We added 100 µL different concentrations of drug-containing medium into 96-well plates, set up a negative control group without drug and positive control group of gentamicin, added 20 µL of bacterial solution to all groups, mixed well, and incubated in 37 °C shaking table for 24 h. Then, we added 20 µL of TTC to each group for 2 h, and took the minimum dilution concentration for a naked eye observation of sterile growth as MIC [17]. On this basis, the sterile growth of the medicated bacterial solution was observed with naked eyes, coated and inoculated on the nutrient agar medium, incubated at 37 °C for 24 h, and the minimum concentration of the drug without bacterial growth in the agar plate was taken as the minimum bactericidal concentration (MBC) [18]. Experiments were performed in triplicate.

#### 3.4.3. Growth Curve

We added 100 µL of the medicated medium with different concentrations into 96-well plates, and added 20 µL of bacterial liquid into each group. After mixing, it was incubated in a 37 °C shaking table for 24 h. We then measured the absorbance at 600 nm every 2 h, and drew the growth curve [19].

#### 3.4.4. Determination of the Release of Nucleic Acids from *S. aureus*

We added the bacterial solution to the drug-containing incubate medium to make the final concentration of the drug 2 MIC, MIC and 1/2 MIC, respectively. The incubate medium without the drug is the control group. It was cultivated at 37 °C for 1 h, then centrifuged at 4000 rpm for 10 min, and the supernatant was collected. The supernatant was then transferred into a fresh tube and then diluted. The amounts of DNA and RNA released from the cytoplasm in supernatant were analyzed by measuring the absorbance at 260 nm [20].

#### 3.4.5. Effect on the Permeability of Bacterial Cell Membrane

The bacterial solution was added to the drug-containing medium to make the final concentration of drugs 2 MIC, MIC and ½ MIC, respectively. The medium without drugs was the control group. It was cultivated at 37 °C for 2 h, centrifuged at 4000 rpm for 10 min, the supernatant was taken, and the extracellular K^+^ content of the bacteria was measured according to the steps of the kit.

#### 3.4.6. Determination of Alkaline Phosphatase (AKP) Activity

The bacterial solution was added to the drug-containing medium to make the final concentration of drugs 2 MIC, MIC and 1/2 MIC, respectively. The medium without drugs was the control group. They were incubated at 37 °C for 2 h, centrifuged at 4000 rpm for 10 min, the supernatant was discarded and resuspended with normal saline occurred. The AKP activity was determined according to the steps of the kit after ultrasonic crushing [21].

#### 3.4.7. Determination of Total ATPase Activity in and out of the Bacteria

The bacterial solution was added into the drug-containing medium to make the final concentration of drugs 4 MIC, 2 MIC, MIC, 1/2 MIC and 1/4MIC, respectively. The medium without drugs was the control group. They were incubated at 37 °C for 30 min, and centrifuged at 4000 rpm for 10 min. The bacterial body and supernatant were stored in ice to prevent ATPase loss. The bacteria were suspended in normal saline, and then placed in ice water bath to break the cells. The total ATPase activity in vivo and in vitro were determined according to the steps of the kit [22].

### 3.5. Determination of Anti-Biofilm Activity

The anti-biofilm activities of EOCM, thymol and carvacrol were measured by crystal violet staining [23]. The drug containing medium with different concentrations was added to a 96-well plate, and the drug free medium was used as the control group. 20 µL of bacterial solution was added to each group, mixed well, and then incubated at 37 °C for 24 h. The bacterial solution was removed, and PBS washed away the floating bacteria. In each group, 100 µL absolute methanol was added to fix for 15 min, the absolute methanol was removed and the medium was dried naturally. In each group, 100 µL crystal violet dye solution was added and dyed for 10 min, PBS cleaned the excess dye solution, and then it was air-dried naturally. Finally, 200 µL 33% acetic acid was added into each hole to dissolve crystal violet, it was shaken and mixed well, and then the absorbance at 600 nm was measured [24].

### 3.6. Scanning Electron Microscope (SEM) Analysis

The bacterial solution and drug solution was added to the 24-well plate to make the final concentration of the drug 2 MIC, MIC, 1/2 MIC and 1/4 MIC. The incubate medium without the drug was the control group. The cell slide was placed in 24-well plates, cultivated at 37 °C for 24 h and the biofilms were then grown on the cell slide. Tweezers were used to carefully remove the cell slide. The incubated solution and floating bacteria were washed with PBS, and fixed with glutaraldehyde solution. Then the fixed solution was washed with PBS, and it was dehydrated with 30%, 50%, 60%, 70%, 80%, 90% and 100% gradient ethanol for 10 min in turn. Observation results of gold spraying scanning electron microscope after natural drying were obtained [25,26].

### 3.7. Confocal Laser Scanning Microscope (CLSM) Analysis

The bacterial solution and drug solution were added onto the confocal plate to make the final concentration of the drug MIC. The biofilm was prepared according to 3.6. The excess bacterial suspension was discarded and wash with PBS. Fluorescent dye was added and the suspensions were maintained in a dark place for 15 minutes. The excess dye was discarded and normal saline was added to maintain the bacterial activity. Results were observed with CLSM [19].

### 3.8. SDS-PAGE Electrophoresis of the Bacterial Proteins

The bacterial solution into the drug containing medium to make the final concentration of the drug MIC. It was cultivated at 37 °C for 24 h, samples were taken 3 h, 6 h, 9 h and 12 h, respectively, it was centrifuged at 4000 rpm for 10 min and bacteria collected. The concentration of the bacterial solution was adjusted with PBS. The protein loading buffer was added, mixed well, added to a boiling water bath for 10 min, centrifuged at 12,000 rpm for 10 min, and the supernatant was taken for SDS-PAGE electrophoresis to detect the effect of the drug on the soluble protein of the bacteria [27].

### 3.9. Antioxidant Activity

#### 3.9.1. Determination of DPPH Radical Scavenging Capacity

Measurements of 2 mL of 1, 5, 10, 15 and 20 mg/mL drug solution and Vc solution, respectively, were taken and mixed with 2 mL of 0.1 mmol/L DPPH free radical solution, then the absorbance Ai at 517 nm was measured after reacting in the dark for 30 min. Absolute ethanol was measured instead of DPPH free radical solution to determine A_j_, and absolute ethanol instead of sample solution A_0_ was used for a blank control to determine absorbance [28,29]. The experiment was conducted in parallel three times, and the results were expressed as average values. The clearance rate of DPPH radical is calculated according to the following formula:

DPPH Radical Scavenging Activity = [1 − (A_i_ − A_j_)/A_0_] × 100%

#### 3.9.2. Determination of Hydroxyl Radical Scavenging Capacity

Measurements of 2 mL of 1, 5, 10, 15 and 20 mg/mL drug solution and Vc solution, respectively, were taken, and add 2 mL of 9 mmol/L FeSO_4_ solution and 9 mmol/L H_2_O_2_ solution were added and mixed well, then stood for 10 min. Then, 2 mL of 9 mmol/L salicylic acid solution was added and mixed well, and stood for 30 min to measure the absorbance Ai at 510 nm. Distilled water instead of salicylic acid solution was used to determine A_j_, and distilled water instead of sample solution A_0_ for blank control [30,31]. The experiment was conducted in parallel three times, and the results were expressed as an average. The scavenging rate of hydroxyl radical can be calculated according to the following formula:

Hydroxyl Radical Scavenging Activity = [1 − (A_i_ − A_j_)/A_0_] × 100%

### 3.10. Statistical Analysis

Statistical analysis was performed using GraphPad Prism. Statistical significance was determined with Student’s *t* test and two-way analysis of variance (ANOVA). *p* values of < 0.05 were considered significant (***, *p* < 0.001; **, *p* < 0.01; *, *p* < 0.05; ns, results no significantly different).

## 4. Conclusions

This experiment analyzed the chemical composition of EOCM through GC-MS. Among them, the type of compounds with the highest proportion was terpenoids and esters, and the main components with a high content are trans-chrysanthenyl acetate (20.23%), aromadendrene (11.47%), thymol (5.69%) and β-caryophyllene (5.26%). Two monomers, thymol and carvacrol, with significant antibacterial activity were screened out through bacteriostasis experiments. Through the preliminary exploration of bacterial mechanism, it was found that EOCM, thymol and carvacrol mainly act on the initial stage of bacterial growth. By destroying the cell membrane, they cause a large amount of material loss in the bacteria, inhibit protein synthesis, affect bacterial metabolic activities, and cannot maintain the normal growth and reproduction of bacteria, thus inhibiting the formation of bacterial biofilm. This experiment also measured the scavenging capacity of EOCM, thymol and carvacrol on DPPH and hydroxyl radicals, but the scavenging capacity of both monomers thymol and carvacrol was weaker than EOCM. The chemical components with strong antioxidant effects need to be explored.

## Figures and Tables

**Table 1:** The chemical component analysis of EOCM.

ID	RT	RI	Compound Name	Molecular Formula	Relative Content (%)	
1	12.317	1100	Linalool	C_10_H_18_O	0.51	(1)
2	14.208	1163	2,4,6-Trimethyl-3-cyclohexene-1-carboxaldehyde	C_10_H_16_O	0.84	(2)
3	14.500	1173	α-Phellandrene-8-ol	C_10_H_16_O	0.14	
4	15.175	1196	α-Terpineol	C_10_H_18_O	0.22	
5	15.767	1218	2-Allyl-6-methylphenol	C_10_H_12_O	0.20	
6	15.992	1227	Nerol	C_10_H_18_O	0.15	
7	16.883	1261	trans-Chrysanthenyl acetate	C_12_H_16_O_2_	20.23	(3)
8	17.433	1283	Carvacrol	C_10_H_14_O	0.52	(4)
9	17.550	1287	Bornyl acetate	C_12_H_20_O_2_	0.29	
10	17.642	1291	Thymol	C_10_H_14_O	5.69	(5)
11	18.908	1353	α-Guaiene	C_15_H_24_	1.00	(6)
12	19.025	1359	1,4,6-trimethyl-5,6-dihydronaphthalene	C_13_H_16_	0.14	
13	19.325	1375	α-Amorphene	C_15_H_24_	0.15	
14	19.442	1381	α-Copaene	C_15_H_24_	1.27	(7)
15	19.575	1388	α-Gurjunene	C_15_H_24_	0.91	(8)
16	19.692	1394	1,2,3,6-Tetramethylbicyclo [2.2.2]octa-2,5-diene	C_12_H_18_	2.79	(9)
17	19.808	1400	Sativen	C_15_H_24_	0.17	
18	19.967	1410	Allo-Aromadendrene	C_15_H_24_	1.20	(10)
19	20.117	1419	Isolongifolene	C_15_H_24_	1.09	(11)
20	20.183	1424	α-Santalene	C_15_H_24_	0.87	(12)
21	20.233	1427	β-Caryophyllene	C_15_H_24_	5.26	(13)
22	20.400	1437	α-Bergamotene	C_15_H_24_	0.23	
23	20.650	1453	β-Santalene	C_15_H_24_	2.29	(14)
24	20.750	1459	Longifolene	C_15_H_24_	0.65	(15)
25	20.825	1464	Humulene	C_15_H_24_	1.83	(16)
26	21.042	1478	2,3,5,6-tetramethyl-Phenol	C_10_H_14_O	2.49	(17)
27	21.092	1481	γ-Cadinene	C_15_H_24_	0.31	
28	21.150	1485	Linalyl isobutyrate	C_14_H_24_O_2_	1.57	(18)
29	21.208	1488	Aromadendrene	C_15_H_24_	11.47	(19)
30	21.342	1497	β-Selinene	C_15_H_24_	0.18	
31	21.433	1503	β-Chamigrene	C_15_H_24_	0.89	(20)
32	21.550	1511	α-Bisabolene	C_15_H_24_	0.21	
33	21.750	1526	Dibenzofuran	C_12_H_8_O	4.24	(21)
34	22.033	1546	Valencene	C_15_H_24_	0.57	(22)
35	22.075	1549	1,1,6-trimethyl-1,2-dihydronaphthalene	C_13_H_16_	0.29	
36	22.167	1556	Camphor	C_10_H_16_O	0.35	
37	22.275	1564	β-Bisabolene	C_15_H_24_	0.55	(23)
38	22.383	1572	Geraniol butyrate	C_13_H_22_O_2_	2.25	(24)
39	22.492	1580	Geranyl isovalerate	C_15_H_26_O_2_	1.66	(25)
40	22.683	1593	β-Caryophyllene oxide	C_15_H_24_O	1.03	(26)
41	22.842	1605	α-Cadinol	C_15_H_26_O	0.23	
42	22.983	1617	Aromadendrene oxide	C_15_H_24_O	0.48	
43	23.058	1623	Cedrenol	C_15_H_24_O	0.47	
44	23.258	1639	Spathulenol	C_15_H_24_O	0.38	
45	23.342	1646	Cedr-8-en-13-ol	C_15_H_24_O	0.35	
46	23.392	1650	Muurolol	C_15_H_26_O	0.33	
47	23.717	1676	δ-Cadinol	C_15_H_26_O	0.30	
48	23.783	1681	LONGICYCLENE	C_15_H_24_	0.59	(27)
49	23.967	1696	Globulol	C_15_H_26_O	0.53	(28)
50	24.017	1700	Pentadecane	C_15_H_32_	0.25	
51	24.317	1726	Valerenal	C_15_H_22_O	0.56	(29)
52	24.508	1743	Cedrol	C_15_H_26_O	0.25	
53	25.625	1843	Hexahydrofarnesyl acetone	C_18_H_36_O	1.28	(30)
54	26.258	1902	d-Nerolidol	C_15_H_26_O	0.54	(31)
55	26.492	1925	Methyl hexadecanoate	C_17_H_34_O_2_	0.59	(32)
56	26.817	1957	neo-Menthol	C_10_H_20_O	1.51	(33)
57	27.433	2018	3-Ethyl-1,2,4,5-tetramethylbenzene	C_12_H_18_	1.05	(34)
58	28.000	2077	Kaur-16-ene	C_20_H_32_	0.53	(35)
59	28.267	2105	4,7-dimethyl-3(2H)-Benzofuranone	C_10_H_10_O_2_	0.49	
60	28.308	2110	Neophytadiene	C_20_H_38_	0.74	(36)
	Total				88.15	

**Table 2:** MIC and MBC of EOCM to *S. aureus*.

Drug	MIC (mg/mL)	MBC (mg/mL)
EOCM	0.625	2.5
Thymol	0.156	0.313
Carvacrol	0.156	0.313

**Table 3:** Antioxidant activity of EOCM, Thymol and Carvacrol.

Drug	IC_50_ DPPH Radical (mg/mL)	IC_50_ Hydroxyl Radical (mg/mL)
EOCM	1.380 ± 0.240	0.712 ± 0.177
Thymol	-	5.882 ± 0.698
Carvacrol	-	4.395 ± 1.339

All values were expressed as Means ± SD (*n* = 3).

## Data Availability

Data will be provided upon request.

## Sample Availability

Samples of EOCM, Thymol and Carvacrol are not available from the authors.
